# Cone Opsins and Inherited Retinal Disease

**DOI:** 10.3390/cells15121098

**Published:** 2026-06-17

**Authors:** Maya Tang, Paul S.-H. Park

**Affiliations:** 1 Department of Nutrition, Case Western Reserve University, Cleveland, OH 44106, USA; 2 Department of Ophthalmology and Visual Sciences, Case Western Reserve University, Cleveland, OH 44106, USA

**Keywords:** cone opsin, inherited retinal disease, deuteranopia, protanopia, tritanopia, photoreceptor cell, mouse models, color blindness

## Abstract

Opsins are the light receptors in retinal rod and cone photoreceptor cells that initiate vision in response to a light stimulus. Rhodopsin is the opsin in rods, and the influence of mutations that disrupt its structure and function has been characterized in detail. Less is known about cone opsins, the opsins in cones, and their gene arrays. Cones are responsible for color vision and high visual acuity and operate under most lighting conditions. There are up to three types of cone opsins (L-, M-, and S-opsin) in most vertebrates, each defined by their distinct spectral sensitivities. Disruptions in the cone opsin gene array cause a variety of inherited retinal disorders, including blue cone monochromacy, Bornholm eye disease, and tritan color vision deficiency. In this review, we discuss what is known about cone opsin mutations and the inherited cone dysfunctions that they cause. We also present the available mouse models that are being used to better understand the pathophysiology promoted by cone opsin defects.

## 1. Introduction

**Figure 1:**
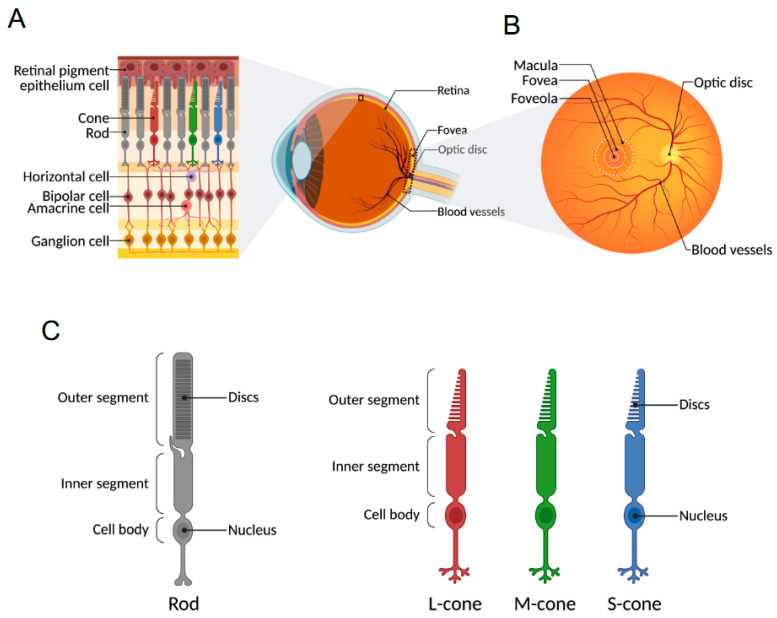
The anatomy of the retina and photoreceptor cell morphology. (**A**). An illustration of a cross-section of the retina in the region of the eye highlighted. The neuronal and retinal pigment epithelium cells of the retina are labeled. (**B**). An illustration of a fundus image in the region of the retina highlighted. The macula, fovea, and foveola are labeled. (**C**). An illustration of rod and L-, M-, and S-cone photoreceptor cells. The outer segment, inner segment, and cell body of photoreceptor cells are highlighted. The discs are present in the outer segment and the nucleus in the cell body. Created in BioRender (https://BioRender.com/5d5k5cc accessed on 9 June 2026).

Vision results from the complex processes in which light signals are transformed into meaningful perception. These processes are carried out by the various neuronal cells found in distinct layers of the retina (Figure 1A). The central retina of humans has a specialized region called the macula, which is required for high visual acuity and is further subdivided into the fovea and foveola (Figure 1B). Vision begins when photoreceptor cells in the outermost layer of the retina absorb and convert light into visual signals that are processed by downstream neuronal cells and are then interpreted by the brain. In mammals, there are two classic light-sensing photoreceptor cells: rods and cones. Rods and cones are compartmentalized into outer segments and inner segments (Figure 1C). The outer segments contain stacked membranous discs that house rhodopsin and cone opsins in rods and cones, respectively.

Rods are the main photoreceptor cells used for scotopic vision (vision under dim-light conditions) [1]. These cells are extremely sensitive, capable of detecting a single photon of light and scattered light [2]. They are useful for the detection of motion but do not contribute to high visual acuity. The development of a photoreceptor cell into a rod cell is dependent on a nuclear receptor (NR2E3) and neural retina-specific leucine zipper protein (NRL) [3,4]. In humans, rods outnumber cones by a ratio of 20:1, but the distribution of rods and cones in the human retina is not uniform [5]. Rod density increases with distance from the central fovea. Having peak rod density in the periphery of the retina is important for dim-light vision and motion detection. Rods still outnumber cones in the macula. Rod density is sparse in the fovea, and rods are completely absent in the foveola, the area of the highest density of photoreceptor cells in the retina [5]. Rods have a long, cylindrical shape, and their outer segments are filled with stacks of membranous discs that are separate from the cell’s plasma membrane (Figure 1C) [6,7]. These discs contain the opsin photopigment, rhodopsin, which is a prototypical G protein-coupled receptor composed of seven transmembrane alpha-helices. Rhodopsin is covalently bound to the chromophore 11-*cis*-retinal in its resting state. The absorption of a photon of light leads to the isomerization of the chromophore to all-*trans*-retinal, which promotes a conformational change in the receptor that then activates the phototransduction cascade [8]. While the structure and function of rods and rhodopsin have been studied extensively, comparatively less is known about cones and cone opsins. Traditionally, rods and rhodopsin have served as useful models to understand the structure and function of cones and cone opsins due to their analogous structural and functional features. However, cones and cone opsins are distinctly tuned for their functional role in the retina [9].

Cones are the main photoreceptor cells used for photopic vision (vision under moderate- to high-light conditions). Cones are the most responsive to direct light. They are important for high visual acuity, spatial resolution, fast light responses, and color vision [1]. In humans, cones are not evenly distributed throughout the retina, and cone density is the highest in the center of the retina in the rod-free foveola, such that about 100,000 cones (out of a total 4.6 million cones) are responsible for the majority of our everyday vision [5]. The preservation of these cones in particular is necessary to prevent blindness. The development of a photoreceptor cell into a cone is dependent on the expression of the nuclear thyroid hormone receptor, THRB/B2 [10,11]. Homodimers or heterodimers of thyroid hormone receptors and retinoid X receptor gamma (RXRG) help to determine cone cell fate and subtype [12]. The cone outer segment is shorter than the rod outer segment, and cone outer segment discs are invaginations of the plasma membrane rather than the free-floating discs in rod outer segments that are distinct from the plasma membrane (Figure 1C) [7,9,13]. This continuity of the cone discs and cone plasma membrane is known as an open disc formation.

Mutations in cone opsins, the photopigments in cone cells that contribute to color vision and high visual acuity, have been recognized as a cause of various inherited retinal diseases. In this review, we will provide an overview of cones and cone opsins, describe the known classes of cone opsin mutations, and enumerate the mouse models available for studying these mutations.

## 2. Cone Photoreceptor Cells and Cone Opsins

**Figure 2:**
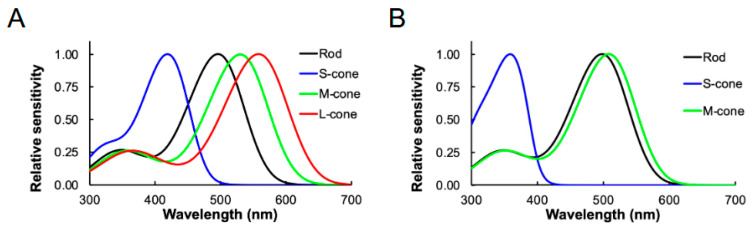
Spectral sensitivity of human (**A**) and murine (**B**) opsins in rod and cone photoreceptor cells. Spectral data kindly provided by Stuart N. Peirson (University of Oxford (Oxford, UK)) based on visual pigment templates with peak sensitivity (λ_max_) values of human and mouse photopigments described previously [20,21,22].

In humans, there are three cone photoreceptor cell subtypes (L-cone, M-cone, and S-cone), defined by the specific cone opsin photopigment expressed, L-opsin (*Opn1lw*), M-opsin (*Opn1mw*), and S-opsin (*Opn1sw*). The distinct sensitivity of these cone opsins to different wavelengths of light forms the basis of human color vision [14]. L-cones allow for the visualization of long wavelengths (red); M-cones, medium wavelengths (green); and S-cones, short wavelengths (blue). The absorption maxima for each human opsin in cone and rod photoreceptor cells are illustrated in Figure 2A. In humans, S-cones are the least populous, representing 8–12% of all cones [15,16,17]. The proportion of L- and M-cones in humans can vary, and there is a wide range of L-:M-cone ratios that still result in normal color vision [18]. There is a higher concentration of L-cones in the periphery, possibly because M-cones develop earlier than L-cones, and the retina develops from the center outwards [19].

**Figure 3:**
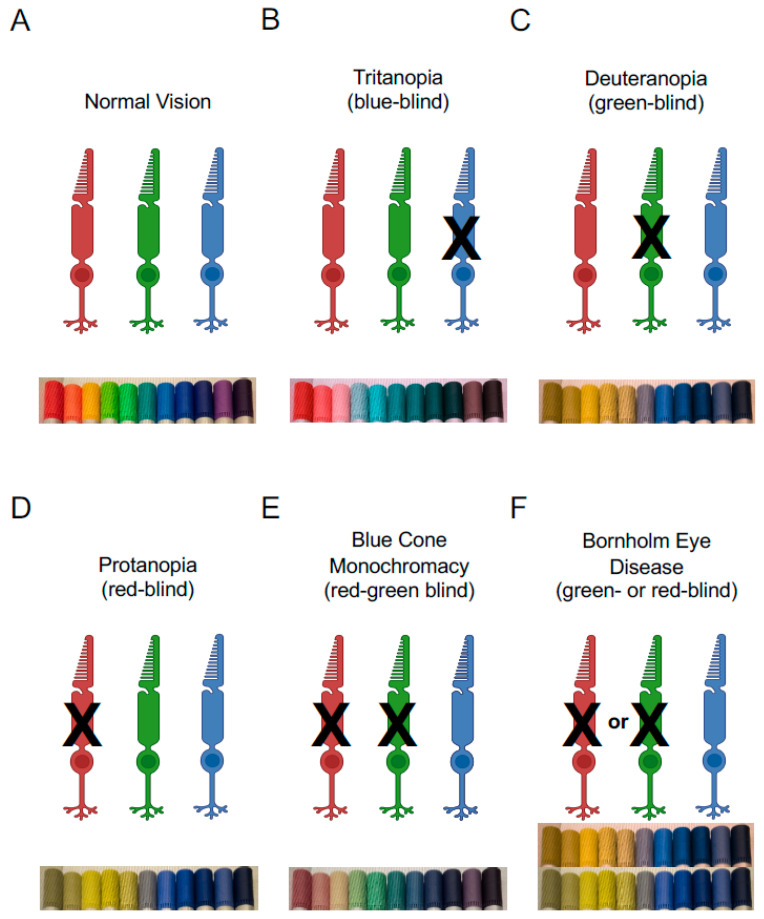
The classification of inherited color vision deficiencies. The color palette available for normal vision (**A**) and a variety of dysfunctional states (**B**–**F**) is shown on the bottom with L-, M-, and S-cones available for vision shown on top (a dysfunctional photoreceptor cell is highlighted with an “X”). The photoreceptor cell images were generated by BioRender.com, and the color palettes were generated by the Coblis color blindness simulator (www.color-blindness.com).

The presence of L-, M-, and S-cones leads to trichromacy (having three independent channels for conveying color signals), and in theory, it can lead to tetrachromacy (four independent channels) in females who can have two different types of L-cones with slightly differing spectral sensitivities [23,24,25,26,27,28]. Tetrachromacy is present in several birds, fish, and reptiles. Trichromacy is present mainly in certain primates and humans, while other mammals tend to be dichromatic [29]. Defects in L-, M-, or S-cones lead to color vision deficiencies (Figure 3).

**Figure 4:**
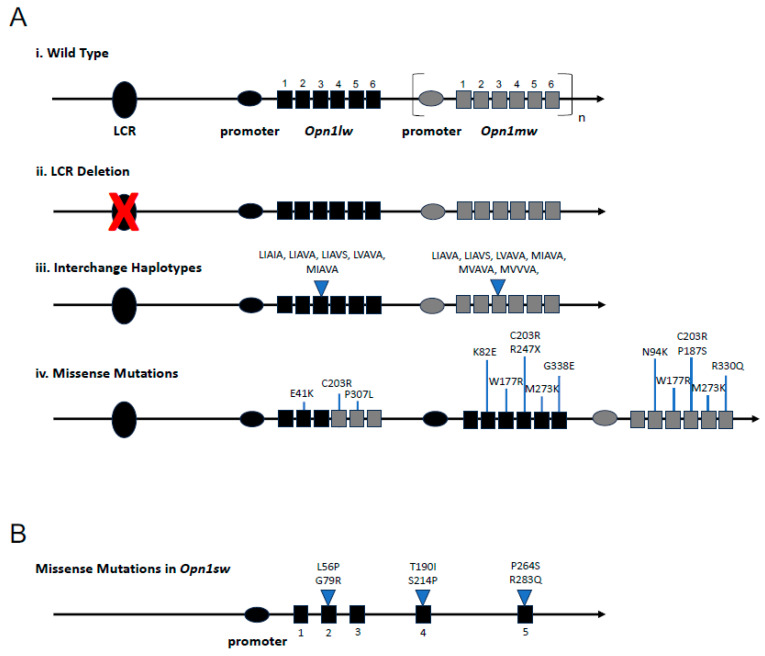
Opsin genetic defects. (**A**). An L/M opsin gene array (**i**) and three classes of pathogenic genetic modifications (**ii**–**iv**). An upstream locus control region (LCR) controls the transcription of the L-opsin (*Opn1lw*) (black) and M-opsin (*Opn1mw*) (gray) genes. Each gene contains 6 exons (black or gray squares) with an upstream promoter. Inherited cone dysfunction can occur due to deletion mutations of the LCR, SNPs in exon 3 and missense mutations in various exons of L-opsin, M-opsin, and hybrid L/M opsin genes. (**B**). Missense mutations in *Opn1sw*. The S-opsin gene comprises 5 exons (black squares) with an upstream promoter. Pathogenic missense mutations are found in various exons of the gene.

The genetics of the opsin gene array lend insight into inherited color vision disorders. The genes for L- and M-opsins, *Opn1lw* and *Opn1mw*, respectively, are located on the X chromosome. The gene for S-opsin, *Opn1sw*, is on an autosome, chromosome 7. *Opn1lw* and *Opn1mw* occur in a tandem array, typically with a single L-opsin gene followed by one or more M-opsin genes (Figure 4A(i)). It is postulated that *Opn1lw* and *Opn1mw* arose from a gene duplication, because they share almost 98% sequence homology [30]. In contrast, both L- and M-opsin share only about 40% sequence homology with S-opsin. Due to their sequence similarity, the L- and M-opsin genes frequently undergo non-homologous recombination and gene conversion, with resultant extra opsin genes, the deletion of whole genes, and hybrid L/M genes. The L- and M-opsin genes each contain six exons. Exons 1 and 6 are essentially identical in L- and M-opsin. Exon 5 contains seven amino acid residue differences between L- and M-opsin, which make the largest contributions to spectral sensitivity differences between L- and M-opsin [30]. Although exon 3 is the most variable exon, only one dimorphism at position 180 alters opsin spectral sensitivity [30,31]. The S-opsin gene only contains five exons and exhibits more sequence differences compared to the exons of L- or M-opsin genes [14].

## 3. Cone Opsin Mutations and Disease

In classical descriptions of color vision deficiencies, the loss or dysfunction of S-cones can result in tritan color vision deficiencies (tritanopia or tritanomaly), loss or dysfunction of M-cones can result in deutan color vision deficiencies (deuteranopia or deuteranomaly), and loss or dysfunction of L-cones can result in protan color vision deficiencies (protanopia or protanomaly). The phenotypic consequences of L- and M-cone dysfunction are typically more complex, however, as recombination events can generate hybrid pigments, altered spectral tuning, reduced opsin expression, or cone degeneration, resulting in a broad spectrum of retinal phenotypes. Several inherited cone dystrophies resulting from mutations in *Opn1lw*, *Opn1mw*, or *Opn1sw* have been recognized [32]. Blue cone monochromacy (BCM) is an X-linked recessive condition caused by the absence of functional L- and M-opsin. Vision is mediated by rods and the remaining S-cones, and hence patients perceive color in shades of blue and gray, with some color discrimination possible depending on lighting conditions (Figure 3E) [33]. Clinically, BCM is characterized by red–green color blindness, as well as myopia, nystagmus, and photophobia, often presenting at birth or in early infancy in affected males [34]. Bornholm eye disease (BED) is an X-linked recessive disorder that was first described in a family in Bornholm, Denmark [35]. Affected males have myopia and either deuteranopia or protanopia (Figure 3F), depending on which opsin gene is affected [36,37]. BED has been shown to be the result of single-nucleotide polymorphisms in exon 3 of either *Opn1lw* or *Opn1mw* [38,39]. Missense mutations in *Opn1sw* lead to tritanopia color vision deficiencies (Figure 3B) that are inherited in an autosomal dominant pattern with incomplete penetrance, affecting both males and females equally [40]. A variety of cone opsin genetic defects can lead to cone dystrophy and include: LCR deletion (Figure 4A(ii)), interchange haplotypes (Figure 4A(iii)), and missense mutations (Figure 4A(iv),B).

### 3.1. LCR Deletion

The transcription of the L- and M-opsin genes is controlled by an upstream locus control region (LCR) and by a promoter element upstream of each of the opsin genes [30,34,41]. The LCR is required for the L- and M-opsin genes to be transcribed. The LCR functions as a long-range enhancer that enables the mutually exclusive expression of a single opsin gene per cone cell [42,43]. Competitive interaction between the LCR and the visual pigment promotors influences which opsin gene will be transcribed. The deletion of the LCR alone or in combination with deletions of some or all the exons of the cone opsin genes can occur [38]. The deletion of the LCR results in a lack of expression of both L- and M-opsin. In addition, the deletion of the LCR results in the shortening of the cone outer segments, reduction in cone density, and disorganization of cone mosaics [44]. LCR deletion results in BCM. Interestingly, although the macula is cone-rich, BCM resulting from LCR deletions is often, but not always, associated with macular degeneration [45]. LCR deletions also result in high myopia, reduced visual acuity, and nystagmus [38].

### 3.2. Interchange Haplotypes

Interchange haplotypes are the result of recombination events between the L- and M-opsin genes in which single-nucleotide polymorphisms (SNPs) occur in exon 3 in unusual combinations that affect gene functioning (Table 1) [46]. These have been abbreviated in the literature by using the single amino acid code at each of the variant SNP positions in exon 3 of the L- and/or M-opsin genes (amino acid positions 151, 153, 155, 171, 174, 178, and 180): LIAIA, LIAVA, LIAVS, LVAVA, MIAVA, MVAVA, and MVVVA. The presence of these SNP combinations results in varying degrees of exon 3 skipping. In other words, when exon 3 contains these SNP combinations, intron splicing is affected, and exon 3 is variably excluded from the resulting spliced mRNA. *Opn1lw* and *Opn1mw* that harbor exon 3 interchange haplotypes either fail to produce a functional photopigment or produce variable degrees of functional photopigment. Vision disorders associated with interchange haplotypes include BCM, red–green color vision deficiencies, high-grade myopia, cone dysfunction and dystrophy, and BED [46]. The phenotypic effect of each of these variants is complicated by the fact that patients may additionally exhibit missense mutations in alternate regions that impact cone opsin structure and function (Table 2).

### 3.3. Missense Mutations

Missense mutations can occur in the coding regions of *Opn1lw*, *Opn1mw* and *Opn1sw* to disrupt the structure and/or function of cone opsins and cause visual dysfunction. The rhodopsin gene is a hotspot for mutations causing retinal disease with well over 100 mutations identified in patients [66,67]. There are comparatively fewer mutations detected in the cone opsin genes, and it is unclear whether the mutations in cone opsins cause disease by mechanisms analogous to those resulting from rhodopsin mutations. Moreover, cone opsin mutations are understudied compared to rhodopsin mutations. A recent study indicates that although mutations in cone opsins may promote similar molecular defects in vitro, the underlying mechanism for visual dysfunction in vivo may be different, possibly due to differences between rods and cones [68]. Inactivating missense mutations in either the L/M-opsin hybrid gene or both L- and M-opsin genes individually cause BCM. Missense mutations that inactivate *Opn1lw* but leave *Opn1mw* functional cause protanopia. Missense mutations that inactivate *Opn1mw* but leave *Opn1lw* functional causes deuteranopia. Missense mutations in *Opn1sw* cause tritanopia.

The most common missense mutation in L- and M-opsin genes is C203R, a substitution of the cysteine at position 203 by arginine [41,58]. This mutation causes BCM, and patients exhibit disruptions of the cone mosaic and thinning of the outer nuclear layer of the retina, indicating cone cell degeneration [59]. The C203R mutation occurs in a cysteine residue analogous to that in rhodopsin at position 187, which is critical for a stabilizing disulfide bridge and causes receptor misfolding and aggregation when mutated [69,70,71,72]. In rhodopsin, the majority of mutations cause misfolding and aggregation, including the common P23H mutation, and aggregation appears to play a role in rod photoreceptor cell loss in vivo [73,74,75]. While in vitro studies indicate that the C203R mutation and the murine ortholog C198R mutation cause M-opsin to mislocalize and aggregate, aggregation does not appear to contribute to cone cell dysfunction in vivo in a knockin mouse model of BCM expressing the murine ortholog C198R M-opsin mutant [68]. Instead, the mutation appears to severely decrease the expression of M-opsin and prevents the formation of outer segments [68,76].

Several other missense mutations affecting either L-, M-, or L/M-opsin hybrid genes have been identified (Table 2) [48,54,55,56,57,60,61,77]. Likewise, several mutations have been identified in *Opn1sw*, resulting in S-cone dysfunction [62,63,64,65] (Figure 4B, Table 2). Patients with *Opn1sw* mutations present with progressive tritanopia; tritan defects may take several decades to progress from mild to moderate deficits [78]. Further characterizations are required for the various mutations identified in all three cone opsin genes to better understand the link between the mutations and their impact on receptor structure/function and cone photoreceptor cell health.

## 4. Mouse Models with Cone Opsin Mutations

Mouse models have served as an invaluable tool in vision research for studying eye disease [79,80]. The rod-dominant murine retina has facilitated studies on the physiology and pathophysiology of rod photoreceptor cells, including those involving rhodopsin and its pathogenic mutants. Significant barriers exist in using mice to study the physiology and pathophysiology of cone photoreceptor cells. There are some important differences between murine and human vision [81,82]. Murine vision is geared towards dim-light (scotopic) conditions, and their retinas are composed primarily of rods. The rods in the murine retina are uniformly distributed, and the retina lacks a fovea, the cone-rich region critical for human vision [83].

Mice possess one rod type and two cone types. Unlike humans, who express three opsin photopigments (L-, M-, and S-opsin), mice are dichromatic and only express M-opsin and S-opsin (Figure 2B), and they lack the L-opsin photopigment. The lack of *Opn1lw* in mice means that the cone opsin gene array in humans is not adequately modeled in mice. Mice, therefore, lack red–green color discrimination. Moreover, a large fraction of cones coexpress M- and S-opsin. True S-cones are concentrated in the ventral retina, which allows them to detect color signals from their upper visual field [84,85]. Humans and mice are similar in terms of the spectral sensitivities of their rods but differ in terms of the spectral sensitivities of their cones (Figure 2), which is determined by the expressed cone opsin. The limitations of the murine retina must be carefully considered when using mouse models to study inherited cone disease. Mouse models still have much value and can provide important insights into human cone disease. We briefly discuss here some of the available mouse models developed to model some aspects of inherited cone disease and test therapeutic strategies.

### 4.1. Mouse Models of Blue Cone Monochromacy

Two types of mouse models have been proposed to model certain aspects of BCM. The first models the deletions of an entire M-opsin gene, the *Opn1mw* knockout mouse [86]. M-opsin expression in these knockout mice is completely absent in the retina. Since mice lack L-cones, these mice only express S-cones, predominantly in the ventral retina. Dorsal cones remain structurally intact but with shortened outer segments, suggesting that despite the lack of M-opsin expression, dorsal cones remain potential targets for recovery if M-opsin was restored, e.g., via gene therapy [86]. Recombinant M-opsin, delivered by adeno-associated virus (AAV), was able to rescue M-cone function in these mice, with the restoration of functional responses to middle-wavelength light stimuli. More recently, the AAV-mediated supplementation of L-opsin in the *Opn1mw* knockout mouse on the all-cone background achieved by *Nrl* knockout also resulted in the structural and functional recovery of cone photoreceptor cells [87]. Together, this provides evidence that gene therapy for BCM patients may be a viable treatment option.

The second BCM model is a knockin mouse expressing the C198R mutation in M-opsin. This mutation is the murine ortholog of the human C203R missense mutation. C198R mutant M-opsin knockin mice have been studied on both the wild-type background and *Opn1sw* knockout background, to eliminate the effect of coexpressed S-opsin in cone photoreceptor cells [68,76]. Both backgrounds exhibited similar phenotypes, including reduced photopic function without impacting rod function, diminished M-opsin expression and the loss of cone outer segments without the loss of the cone photoreceptor cell itself. The viability of cone photoreceptor cells in this model also suggested the possibility of gene therapy approaches to reintroduce functional cone opsins. Indeed, gene supplementation by an AAV vector carrying either *Opn1mw* or *Opn1lw* rescued cone structure and function in this mouse model, supporting the potential for gene therapy approaches to combat BCM [76]. The rescue effect was much more pronounced in mice treated at 1 month of age, compared to mice treated at 5 months of age, indicating that gene therapy must be delivered early to achieve the maximal functional and structural recovery of cones.

### 4.2. Mouse Models of Bornholm Eye Disease

Knockin mouse models have been developed to study exon 3 interchange haplotypes associated with BED [35,38,39,50,52], a disease characterized by high myopia and deuteranopia in males [35]. In these mice, portions of the M-opsin gene were replaced with sequences from the human L-opsin gene containing exon 3 interchange haplotype variants. Mouse models with three different haplotypes were generated, LIAIS (*Opn1lw*^LIAIS^), LVAVA (*Opn1lw*^LVAVA^), and LIAVA (*Opn1lw*^LIAVA^) [88,89]. To avoid complications associated with the dual expression of cone opsins occurring in single murine cone photoreceptor cells, knockin mice were on an *Opn1sw* knockout background, allowing for the sole expression of the mutant L-opsin knockin gene. Of the three models, *Opn1lw*^LVAVA^ mice exhibit the greatest similarity to the human BED phenotype, which is milder than the BCM phenotype [89]. *Opn1lw*^LVAVA^ mice produce a partially functional L-opsin protein, but they induce cellular stress and reduced cone function. Studies from these mouse models illustrate that BED may arise from multiple genetic variations and that the BED phenotype likely arises from a combination of altered protein function and defective RNA processing.

## 5. Concluding Remarks

In this review, we discussed the known mutations in *Opn1lw*, *Opn1mw*, and *Opn1sw*, the genes coding for L-, M-, and S-opsin. These mutations lead to inherited color vision diseases, specifically, BCM (L- and M-opsin), BED (L- and M-opsin), and tritan color vision deficiency (S-opsin). Although humans and mice have important differences in their visual systems, mouse models of human disease have provided useful information on the pathophysiology promoted by cone opsin defects. Furthermore, mouse models are proving to be useful tools for the advancement of gene therapy techniques that may soon benefit patients with inherited color vision deficiencies.

## Figures and Tables

**Table 1:** Exon 3 haplotypes.

Haplotype	Opsin Affected	Phenotype	References
LIAIA	L	Myopia; color confusion; reduced photopic ERG	[47]
LIAVA	L, M	Moderate to high myopia; protanopia, astigmatism; reduced cone function; reduced visual acuity	[38,46,48,49,50,51]
LIAVS	L, M	Myopia; deuteranopia if LIAVS and no other opsin gene or second opsin with LIAVA; reduced photopic ERG; poor visual acuity	[46,51]
LVAVA	L, M	Myopia; in isolation, no red–green color vision defect; in combination with defects in other genes in the array, can range from normal color vision to protanopia to deuteranopia; reduced cone function; reduced visual acuity	[38,46,52]
MIAVA	L, M	Myopia, deuteranopia or protanopia depending on combination defects in gene array; reduced cone function; reduced photopic ERG	[38,46,47]
MVAVA	M	Severe myopia; normal red–green color vision; relatively preserved visual acuity	[46,47,53]
MVVVA	M	In isolation, normal visual acuity, normal color vision, normal cone function; combined with other mutations (e.g., LIAVA, LVAVA) can increase disease severity	[38,47]

**Table 2:** Missense mutations in L-, M-, and S-opsin.

Mutation	Opsin Affected	Phenotype	References
E41K	Hybrid L/M	Myopia; cone dysfunction; protanopia; photophobia; astigmatism	[54]
K82E	L	Decreased visual acuity; poor night vision; poor color discrimination; cone dysfunction	[55]
N94K	M	Deuteranomaly	[56]
W177R	L, M	Myopia; reduced visual acuity; nystagmus; macular atrophy; reduced photopic ERG; blue cone monochromacy-like color vision (functioning S-cones; non-functional L-, M-cones)	[57]
P187S	M	Deuteranopia	[48]
C203R	L, M, hybrid L/M	Myopia; nystagmus; Severe protan and deutan defects with preserved tritan discrimination; severe cone dysfunction; loss of central foveal photoreceptor structure; variable macular atrophy	[38,41,58,59]
R247X	L	Myopia; reduced visual acuity; nystagmus; photophobia; severe protan and deutan defects, with preserved tritan discrimination; progressive thinning of outer nuclear layer of foveola	[60]
M273K	L, M	Myopia; reduced visual acuity; nystagmus; photophobia; severe protan and deutan defects, with preserved tritan discrimination	[48,61]
P307L	Hybrid L/M	Myopia; reduced visual acuity; nystagmus; photophobia; severe protan and deutan defects, with preserved tritan discrimination	[60]
R330Q	M	Deuteranopia	[56]
G338E	L	Protanopia	[56]
L56P	S	Tritanopia	[62]
G79R	S	Tritanopia	[63]
T190I	S	Mild tritanopia	[64]
S214P	S	Tritanopia	[40]
P264S	S	Tritanopia	[63]
R283Q	S	Tritanopia; progressive disruption in cone mosaic over time	[65]

## Data Availability

No new data were created or analyzed in this study.
